# The Fetus as a Cardiac Patient: Assessment and Therapy of Cardiovascular Pathology before Birth

**DOI:** 10.1155/2010/974520

**Published:** 2010-12-29

**Authors:** Anita J. Moon-Grady, Shinjiro Hirose, Greg Kesby, Samuel Menahem, Wayne Tworetzky

**Affiliations:** ^1^Fetal Treatment Center, University of California San Francisco, CA 94143, USA; ^2^Department of High Risk Obstetrics and Fetal Medicine, Royal Prince Alfred Hospital, Sydney, NSW 2050, Australia; ^3^Fetal Cardiac Unit and Department of Pediatrics, Monash Medical Centre, Melbourne, VIC 3168, Australia; ^4^Advanced Fetal Care Center, Children's Hospital Boston, Boston, MA 02115, USA

The past few decades have seen major advances in evaluation and treatment of fetal cardiovascular diseases. Largely due to advances in imaging, recognition of structural pathology in the developing human heart can now be performed as early as the 12th week of pregnancy and can be seen to develop and progress through gestation. Because of the observation that serious structural congenital heart disease may progress from seemingly minor disease if untreated, several centers are now intervening before birth to address such abnormalities and attempt to prevent the further development of structural disease. Furthermore, detailed assessment of cardiac rhythm, function, and myocardial mechanics is now also possible as early as the first trimester. Transplacental treatment for fetal rhythm abnormalities has dramatically changed the outcomes for affected pregnancies in the past decade. More recently, several centers have begun to incorporate routine fetal cardiovascular assessment in the evaluation of diseases such as congenital cystic adenomatiod malformation of the lung, twin-twin transfusion syndrome, and congenital diaphragmatic hernia where structural disease may impose significant comorbidity postnatally, and hemodynamic derangements and functional pathology secondary to the primary process may impact the fetus in utero. Recognition of potentially treatable fetal cardiac disease may alter the prognosis for these patients in the perinatal period as in utero treatment to address the primary abnormality has been shown to improve the hemodynamic derangements observed. Finally, prenatal recognition of fetal cardiac disease in general may be changing the natural history and incidence of disease in the postnatal population. 

Regardless of training and background, any healthcare professional involved in the diagnosis and management of diseases of the fetus and newborn now needs to be cognizant of the potential contribution of prenatal cardiac assessment and treatment in the congenitally malformed or unwell fetus. In this special issue on the fetus as a cardiac patient, we have invited a few papers addressing issues unique to this patient group.

The first paper of this special issue addresses ethical issues relating to fetal diagnosis of a major abnormality with special emphasis on cardiac malformations. Presented are discussions of the ethical concept of beneficence and the principle of patient autonomy in the context of counseling expectant mothers and the complex ethical situation which arises in the consideration of the fetus as a patient.

The second paper presents a comprehensive review of cardiac findings in twin-to-twin transfusion syndrome (TTTS), a condition which is a severe complication of monochorionic twin pregnancy. TTTS is characterized clinically on ultrasound by polyhydramnios in the “recipient” twin and oligohydramnios in the “donor” with varying degrees of cardiac dysfunction in the recipient. The pathophysiology of the syndrome itself and of the development of cardiomyopathic changes remains incompletely understood. The review discusses what is known with respect to the cardiac findings at presentation, natural history, and response to treatment and discusses current approaches to a comprehensive cardiac evaluation of affected fetuses. The third paper describes a large series of fetuses presenting with findings consistent with cardiomyopathy or myocarditis and represents a large natural history study of these entities, underlining the particularly high perinatal loss rate with diagnosis of dilated cardiomyopathy or myocarditis, as opposed to many of the hypertrophic myopathies.

The issue concludes with two thought-provoking review articles regarding the intrauterine environment and the complex interaction the developing fetal brain and circulatory system have with each other and the placental circulation. In the first of these, the authors present a discussion of intrauterine hypoxia, its various causes, and mechanisms whereby disease in the fetus may result. The final paper presents a comprehensive review of the current understanding of pathologic findings in the developing brain of the fetus and infant with congenital heart disease. Methods for detection, potential etiologies, and implications for neurodevelopmental outcome are discussed. Intriguing speculation regarding the possibility of altering the natural history of developmental brain abnormalities via in utero intervention will leave the reader eagerly anticipating future developments in the rapidly advancing field of fetal cardiovascular assessment and treatment. 

*Anita J. Moon-Grady* *Anita J. Moon-Grady* *Shinjiro Hirose* *Shinjiro Hirose* *Greg Kesby* *Greg Kesby* *Samuel Menahem* *Samuel Menahem* *Wayne Tworetzky* *Wayne Tworetzky*

